# Central Pontine Myelinolysis and Hypokalemic Paralysis as Presenting Manifestations of Sjogren’s Syndrome

**DOI:** 10.7759/cureus.45233

**Published:** 2023-09-14

**Authors:** Ashraf V V, Sajith Narayanan, Remesh Bhasi, Ramakrishnan KG

**Affiliations:** 1 Neurology, Aster Malabar Institute of Medical Sciences (MIMS) Hospital, Calicut, IND; 2 Nephrology, Aster Malabar Institute of Medical Sciences (MIMS) Hospital, Calicut, IND; 3 Rheumatology, Aster Malabar Institute of Medical Sciences (MIMS) Hospital, Calicut, IND; 4 Radiology, Aster Malabar Institute of Medical Sciences (MIMS) Hospital, Calicut, IND

**Keywords:** trident sign, sjogren’s syndrome, renal tubular acidosis, hypokalemia, central pontine myelinolysis

## Abstract

Neurological involvement in Sjogren’s syndrome can have varied manifestations and can precede the classical sicca symptoms of Sjogren’s syndrome. A 32-year-old woman presented with acute quadriparesis and dysarthria. She had severe hypokalemia, and an MRI of the brain showed a lesion in the central pons that was hyperintense on T2 and fluid-attenuated inversion recovery (FLAIR) sequences sparing the periphery, a trident appearance characteristic of central pontine myelinolysis (CPM). On further evaluation, she was found to have distal renal tubular acidosis (dRTA) due to primary Sjogren’s syndrome. She was treated with steroids and other supportive measures, and she recovered completely in one month. We describe a mild form of CPM with classical MRI features in a patient with Sjogren’s syndrome and hypokalemia due to dRTA.

## Introduction

Sjogren syndrome (SS) is a chronic autoimmune disease characterized by lymphocytic infiltration of the exocrine glands. Common presenting symptoms include dry eye, dry mouth (sicca symptoms), and polyarthralgia. In the course of SS, many other internal organ systems like the kidneys, liver, joints, skin, and nervous system may be involved [1]. The most common neurological complication is peripheral neuropathy [2]. Central nervous system involvement is less common [3]. The nervous system can also be affected by electrolyte abnormalities like hypokalemia or hypernatremia secondary to renal involvement. We report two unusual presenting manifestations affecting the nervous system in a patient with Sjogren’s syndrome: hypokalemic paralysis and central pontine myelinolysis.

## Case presentation

A 32-year-old woman presented with a history of general malaise for seven days and weakness of the limbs for two days. She felt severe tiredness a week prior and took a few vitamin tablets for it. She developed weakness in the upper and lower limbs two days prior, which was of insidious onset and progressed over the next two days, and she became bedbound by the time she reached our emergency department. There was no history of altered sensorium, seizures, lower urinary tract symptoms, or bowel symptoms. Her medical history was positive for hypothyroidism detected two months ago, and she also reported increased thirst for almost a year.

On physical examination, vital signs were within normal limits, and higher mental functions were intact except for mild dysarthria. Motor power was graded 1/5 in the lower limbs and 2/5 in the upper limbs, affecting both the proximal and distal groups of muscles. All deep tendon reflexes were sluggish, and limbs were flaccid. There were no sensory deficits, and the cranial nerve examination was unremarkable.

**Figure 1:**
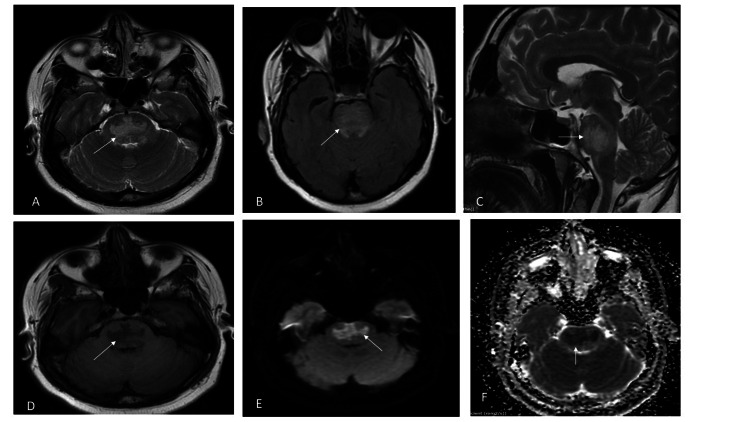
An MRI of the brain demonstrates a trident sign. The T2 axial (A), T2 FLAIR axial (B), and T2 FLAIR sagittal (C) images show a hyperintense signal in a trident shape involving the central pons (arrows). The T1 axial sequence (D) shows a hypointense signal in the pons (arrows). Diffusion-weighted and apparent diffusion coefficient sequences (E and F, respectively) show diffusion restriction in a trident shape involving the central pons (arrow). FLAIR: fluid-attenuated inversion recovery

Her hemoglobin was 12 g/dl, her total leucocyte count was 5200/microliter, her platelet count was 2.2 x 109/L, her erythrocyte sedimentation rate was 38 mm/hour, and her C-reactive protein was 15 mg/dL. Biochemical parameters revealed low serum potassium of 1.7 mmol/L, normal serum sodium of 145 mmol/L, and mildly raised serum creatine phosphokinase of 415 IU. Other blood investigations revealed chloride 113 mmol/L, calcium 10 mg/100 ml, magnesium 3.8 mg/dL, and thyroid stimulating hormone 2.9 microIU/ml. Blood sugar, liver function tests, and renal function tests were within normal limits. Therefore, a diagnosis of hypokalemic paralysis was made, and potassium correction was initiated with IV potassium. Arterial blood gases showed a bicarbonate level of 10 meq/L and a partial pressure of carbon dioxide (pCO_2_) of 24 mmHg. Serum osmolality was 299 mOsm/L, and urine osmolality was 261 mOsm/L. The patient had non-anion gap metabolic acidosis. Antinuclear antibody was positive; anti-Ro/Sjogren syndrome antigen A (SSA) and anti-La/SSB were strongly positive. The Schirmer test was positive for dry eyes. Hypokalemia was corrected, and her limb weakness improved in 48 hours, but she continued to have dysarthria. An MRI of the brain was done, which showed hyperintensity in the central pons sparing the periphery on T2-weighted and fluid-attenuated inversion recovery (FLAIR) images and diffusion-weighted sequences (trident appearance). It appeared hypointense on T1-weighted and apparent diffusion. Coefficient sequences with no evidence of significant enhancement (Figure 1) were consistent with central pontine myelinolysis (CPM).

She was treated with intravenous methylprednisolone, followed by oral steroids. Her neurological symptoms completely resolved in one month. A repeat MRI done after two months revealed a small area of gliosis in the central pons. She was maintained on low-dose oral steroids and hydroxychloroquine.

## Discussion

The nervous system and the kidneys are the two most common organ systems affected in primary SS. Available literature data estimate the prevalence of neurological symptoms in about 8.5%-60% of patients diagnosed with SS [1,2]. Sjogren’s syndrome commonly affects the peripheral nervous system, and central nervous system involvement is much less common. Our patient had two nervous system complications, hypokalemic paralysis, and CPM, at presentation, not due to direct lymphocyte infiltration of the nervous system but due to electrolyte abnormalities. Various electrolyte abnormalities like hypokalemia, hypernatremia, and hyponatremia can occur during the course of SS or at presentation [2].

Our patient presented with muscle weakness secondary to severe hypokalemia, and on further evaluation, she had normal anion gap metabolic acidosis. These findings were suggestive of hypokalemia due to distal renal tubular acidosis (distal RTA). Further history obtained from the patient revealed foreign body sensations in the eyes and dry mouth for the past 18 months, which prompted us to evaluate the possibility of SS. The two common forms of renal involvement in SS are interstitial nephritis and distal renal tubular acidosis. Other rare renal manifestations include glomerular diseases like membranoproliferative glomerulonephritis (MPGN) and membranous nephropathy (MN) [4]. Hypokalemia is the most common electrolyte abnormality in patients with RTA. The causes of hypokalemia include decreased distal tubular sodium (Na+) delivery, secondary hyperaldosteronism, defective hydrogen potassium ATPase (H+-K+ ATPase), and bicarbonaturia [5]. Severe hypokalemia leads to myopathy, as in this patient. A decrease in extracellular potassium concentration causes the transfer of intracellular potassium to the extracellular space, which will further imbalance the sodium-potassium pump and lead to cell edema and degeneration. This causes an increase in membrane permeability and the release of intracellular creatine kinase (CK) into the blood [6]. Hypokalemic paralysis as a presenting manifestation of SS is extremely rare [4,5,6].

Our patient had persistent dysarthria despite the correction of hypokalemia, which prompted us to do an MRI brain evaluation. The MRI brain revealed the classic trident-shaped abnormality in the pons( best on T2 weighted and diffusion sequences) on axial images (or omega sign), which is highly suggestive of CPM. This trident shape is due to heterogenous myelinolysis, which spares the ventrolateral longitudinal fibers and corticospinal tracts [7]. Central pontine myelinolysis is a non-inflammatory demyelination in the central basis pontis. In about 10%-15% of patients with CPM, demyelination also affects extrapontine regions, including the basal ganglia, thalamus, and midbrain. Classical clinical features include impaired consciousness, mutism, and spastic quadriparesis [8]. However, this patient had mild dysarthria without any of the above classical features. With recent advances in neuroimaging techniques such as MRI, cases of asymptomatic or minimally symptomatic CPM have been reported [8]. Regarding the pathogenesis of CPM, abnormalities in serum electrolytes and serum osmolality have mainly attracted attention. The disruption of the blood-brain barrier occurring secondary to osmotic stress is thought to be one of the leading factors in the pathogenesis of CPM. Rapid correction of hyponatremia, mostly an increase in sodium concentration >18 meq/dL in the first 48 hours, is the most common setting for the development of CPM [8,9].

However, other electrolyte abnormalities have also rarely been associated with CPM. Correction of hypernatremia can rarely cause CPM [9]. Recently, severe hypokalemia without any other electrolyte abnormalities causing CPM has also been reported [10,11]. Most of these patients had underlying diseases like diabetes mellitus, anorexia nervosa, chronic alcoholism, and hypoparathyroidism [12]. There are few case reports of patients with SS who developed CPM with hypokalemia and/or hypernatremia [13,14,15]. Hypokalemia causing nephrogenic diabetes insipidus, followed by a rapid rise in sodium resulting in osmotic stress, could potentially explain the occurrence of CPM in SS [16]. Most of the reported cases of CPM in SS patients had a good recovery compared to CPM due to other causes. This raises the question of immune mechanisms in the pathogenesis of CPM in patients with SS [17]. A recent review of osmotic demyelination in Sjogren’s syndrome by Sandhya et al. postulated defective functions of aquaporins as a possible mechanism [17].

## Conclusions

This case highlights two rare presenting manifestations of Sjogren’s syndrome, hypokalemic paralysis, and CPM. When patients with primary SS present with these manifestations, it might create diagnostic confusion. In the setting of SS with RTA, CPM must be suspected when a patient with flaccid weakness does not respond to the correction of hypokalemia or develops additional neurological features.
